# Anticoagulants for the Control of the Common Vampire Bat (*Desmodus rotundus*)

**DOI:** 10.1111/zph.13196

**Published:** 2024-12-22

**Authors:** Laura Ávila‐Vargas, Diego Soler‐Tovar, Quan Dong, Luis E. Escobar

**Affiliations:** ^1^ Semillero de Investigación Una Salud, Facultad de Ciencias Agropecuarias Universidad de La Salle Bogotá Colombia; ^2^ Grupo Epidemiología y Salud Pública, Facultad de Ciencias Agropecuarias Universidad de La Salle Bogotá Colombia; ^3^ Department of Fish and Wildlife Conservation Virginia Tech Blacksburg Virginia USA; ^4^ Global Change Center Virginia Tech Blacksburg Virginia USA; ^5^ Center for Emerging Zoonotic and Arthropod‐Borne Pathogens Virginia Tech Blacksburg Virginia USA; ^6^ Kellogg Center for Philosophy, Politics, and Economics Virginia Tech Blacksburg Virginia USA

**Keywords:** anticoagulant, bats, *Desmodus rotundus*, rabies, vampire bat

## Abstract

**Background:**

In Latin America, there is a high incidence of vampire bat‐transmitted rabies in cattle causing increased mortality of livestock, which heavily impacts the agricultural sector. Anticoagulants‐based control methods for the common vampire bat ( *Desmodus rotundus* ) have been employed continuously since the 1970s with various methods of application, presentations, doses and active ingredients. Studies from half a century ago still serve as a reference for the current use of anticoagulants for bat‐borne rabies control in Latin America. The objective of this study was to structurally and bibliometrically review literature on the use of anticoagulants for the control of *D. rotundus* as a means of rabies control.

**Materials & Methods:**

Scientific literature on the use of anticoagulant products for *D. rotundus* control was obtained, reviewed and analysed. Articles were retrieved from Scopus and Web of Science databases. Research articles from 1971 to 2021 in Spanish, English and Portuguese were included in the review. Results were visualised using RStudio, Bibliometrix and VOSviewer.

**Results:**

The body of literature indicates effectiveness of up to 100% in the use of anticoagulants to induce bat mortality. The effectiveness of anticoagulants for rabies control, however, remains uncertain. No evidence was found to support or refute the use of anticoagulants for rabies control.

**Discussion:**

Instead, literature suggests that disturbing bat colonies increases rabies prevalence. This finding suggests that anticoagulants may have the opposite intended effect on rabies control and highlights the importance of further research on the practical methods for bat‐borne rabies prevention.

**Conclusion:**

Field experimental studies that include control groups over areas and periods that account for *D. rotundus* ecology are needed to determine the effectiveness of anticoagulants for rabies control in livestock. In conclusion, the use of anticoagulants for rabies control is questionable.

**Summary**

- The use of anticoagulants for vampire bat control, while historically effective in reducing bat populations, does not provide conclusive evidence of their efficacy in preventing rabies virus transmission.
- This literature review highlights the lack of recent studies that adequately evaluate the efficacy (or lack thereof) of anticoagulants in the control of bat‐borne rabies in cattle.
- Previous literature suggests that disturbing bat colonies in general may be counterproductive in preventing rabies transmission.

## 1 Introduction

Rabies virus (RABV) is a prevalent veterinary and public health threat in Latin America (Sánchez et al. 2019; Mantari Torpoco et al. 2019; Frantchez and Medina 2018). The most common RABV strain is a *Lyssavirus* in the viral family Rhabdoviridae. Typical transmission of RABV is through direct physical contact between the saliva of an infected animal and an open orifice or wound of a susceptible individual causing a mortality rate of nearly 100% in both humans and animals (OMSA 2023; Scheffer et al. 2014). In the Americas, sanguivorous bats (i.e., bats that feed exclusively on blood), commonly known as vampire bats, are an important reservoir of RABV because they transmit the virus through bites when they feed on their prey (Wallau et al. 2023; Pradilla Ardila 2010; Fisher, Streicker, and Schnell 2018; Bárcenas‐Reyes et al. 2015). There are three species of vampire bats, including *Desmodus rotundus* , *Diaemus youngi* and *Diphylla ecaudata* (Quintana and Pacheco 2007; Greenhall, Joermann, and Schmidt 1983). Among vampire bat species, only *D. rotundus* feeds primarily on mammals, especially large mammals such as cattle, while *D. youngi* and *D. ecaudata* feed primarily on birds (Quintana and Pacheco 2007; Greenhall, Joermann, and Schmidt 1983; Cárdenas‐Canales et al. 2022).

*Desmodus rotundus* is a nocturnal predator that is able to consume 7.5 L of blood per year per individual bat (Bhatnagar 2008). *Desmodus rotundus* individuals tend to feed every night on the same or new prey individuals, ingesting ~20 mm of blood a night (Greenhall 1971; Flores‐Crespo, Burns, and Said Fernández 1974). *Desmodus rotundus* has specialised teeth designed for lacerating the skin, which facilitates continuous bleeding wounds (Mollerach and Mangione 2004). These bites result in chronic, open wounds that are susceptible to secondary bacterial, fungal and parasitic infections (Flores‐Crespo 1978; Acha 1967; Arellano‐Sota, Sureau, and Greenhall 1971). Animals preyed upon by *D. rotundus* suffer significant blood loss, increasing susceptibility of metabolic conditions like anaemia, weight loss and decreased meat and milk production (Flores‐Crespo 1978; Greenhall 1971; Acha 1967; Arellano‐Sota, Sureau, and Greenhall 1971).

In addition to causing chronic and susceptible wounds, *D. rotundus* bites could transmit RABV and result in the death of cattle, presenting a potential zoonotic risk to humans. This impact was further exacerbated in Latin America in the 1970s, when countries expanded cattle ranching, providing an opportunity of a dietary shift for *D. rotundus* towards livestock (Lopes, Moraes, and Wilcox 2020; Cavallotti Vázquez 2006; Voigt and Kelm 2006; Anderson et al. 2014; Kraker‐Castañeda and Echeverría‐Tello 2012; Johnson, Aréchiga‐Ceballos, and Aguilar‐Setien 2014). As a result, human aversion towards *D. rotundus* grew and various control measures were implemented to combat vampire bats. *Desmodus rotundus* control methods included the use of lanterns, nets, protective grids and burning of roosts. Additionally, insecticides such as toxaphene, hydrocarbons such as diesel and the use of explosives and dynamite were used in caves (Flores‐Crespo 1978; Greenhall 1971). These population control methods contributed to the decline of local vampire bat populations and other non‐sanguivorus bat species populations, which contribute to fundamental ecosystem services such as pest control, pollination and seed dispersal (Greenhall 1971).

Researchers in the 1970s were motivated to develop a better understanding of *D. rotundus* life history and found that this species preens individually and communally for 2–3 h per day (Linhart, Flores‐Crespo, and Mitchell 1972). Using this grooming behaviour, population control strategies have been developed based on applying a mixture of toxic compounds and a viscous vehicle (López García et al. 2015). The toxic paste is applied to the back of a *D. rotundus* individual, which is expected to disperse the compound to ~20 individuals (Linhart, Flores‐Crespo, and Mitchell 1972). In 1971, researchers found that *D. rotundus* moved frequently between roosts, facilitating active dispersal of population control interventions (Mitchell et al. 1972). Nevertheless, recent studies suggest that movement between roosts may not be as common as previously thought. When *D. rotundus* individuals do move, it appears to be primarily sex‐motivated, with young males tending to change locations more frequently than females (Delpietro et al. 2017; Streicker, Recuenco, et al. 2012).

In the early 1970s, several compounds were evaluated for the control of *D. rotundus* , including organophosphates such as Warbex (famfur active ingredient) and Cygon (dimethoate active ingredient), with only Warbex (famfur) producing the expected mortality in treated individuals (Said 1973). Rodenticides were also evaluated, which are anticoagulant poisons that cause death by haemorrhaging in exposed individuals. For example, chlorophacinone produced a 95% mortality rate in treated *D. rotundus* (Linhart, Flores‐Crespo, and Mitchell 1972). Diphenadione showed some toxicity as evidenced by visible signs of anticoagulant poisoning in *D. rotundus* individuals (Said 1973). Warfarin, on the other hand, proved to be highly effective with a 100% mortality rate in treated individuals (Flores‐Crespo et al. 1979).

In addition to evaluating anticoagulant poison effects on *D. rotundus* individuals, application techniques were tested on cattle afflicted by bites. Anticoagulant compounds were applied to cattle wounds, achieving an 80% reduction in bites and afflicted animals (Flores‐Crespo et al. 1979). Injectable methods in cattle, both intraruminal and intramuscular, were effective in reducing *D. rotundus* bites, although with short‐lasting effects (Flores‐Crespo et al. 1979). These effective vampire bat‐specific anticoagulants, called ‘vampiricids’, have seen widespread, continuous use in the Latin American livestock industry since their implementation.

Currently, RABV transmitted by *D. rotundus* has surpassed RABV transmitted by dogs in Latin America. Infected *D. rotundus* bats often prey on domestic animals such as dogs and cats, contributing to the spread of the RABV virus (Rocke, Streicker, and Leon 2023). As a result, human rabies cases in Latin America are now predominantly associated with bat transmission, making bat‐borne RABV an emerging problem (Rocke, Streicker, and Leon 2023).

The objective of this study was to assess the use of anticoagulants called vampiricids for the control of *D. rotundus* and RABV based on the review and analysis of the results obtained from scientific literature. Bibliometric data were collected for the types of studies conducted, active ingredients used, methods of application, results obtained and effect on the epidemiology of rabies. Results from this analysis may help guide future research on RABV control efforts in the Americas.

## 2 Materials and Methods

**FIGURE 1:**
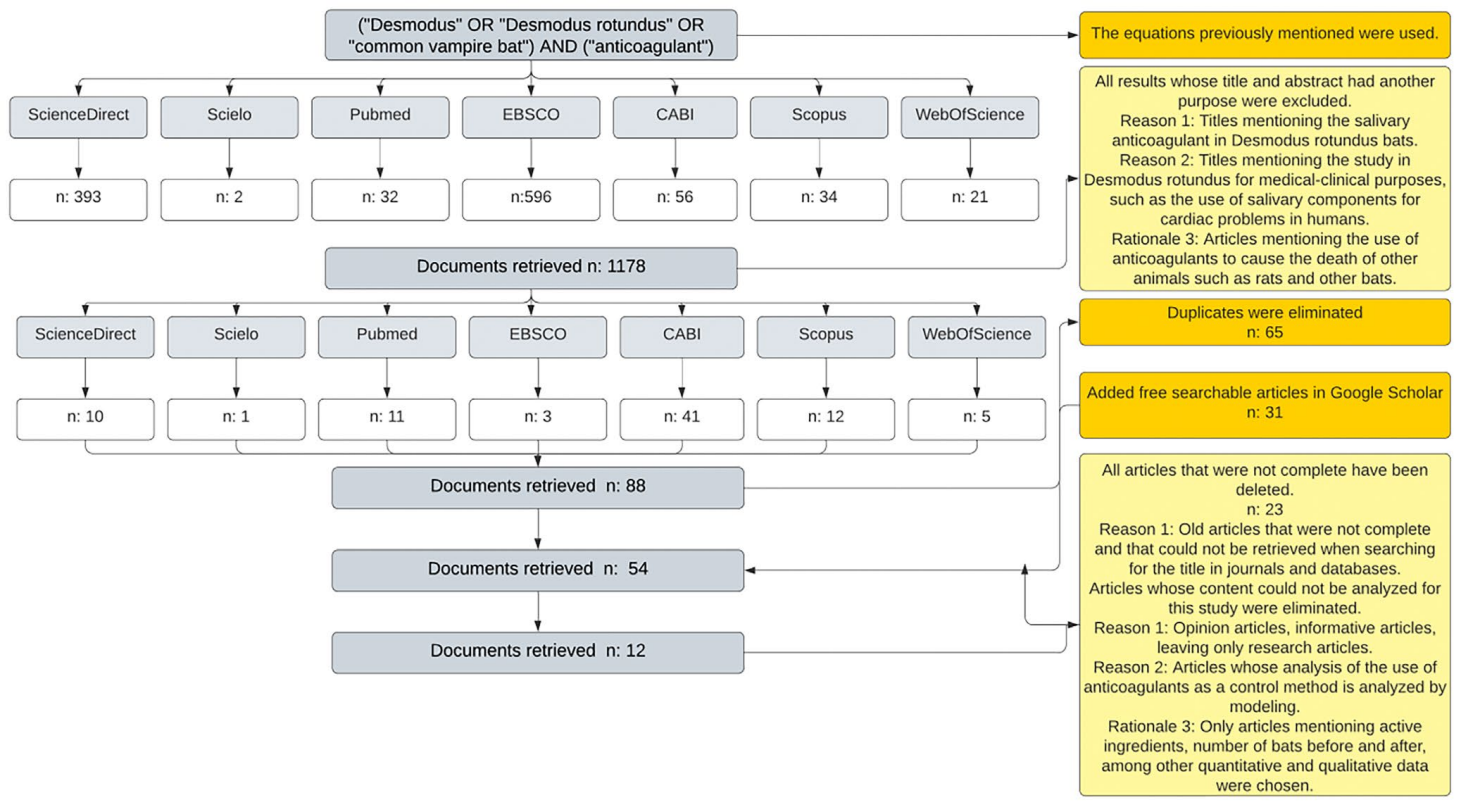
Flowchart used to collect, screen and retain articles for this review. The diagram should be read from top to bottom, while following the direction of the arrows. The grey boxes represent the databases consulted (e.g., Scopus), while the white boxes indicate the number of results obtained in each database (*n*). The yellow boxes on the left indicate the exclusion criteria applied. The black arrows with triangular tips indicate the successive reduction of the number of documents retrieved.

A total of 1178 publications were evaluated following a structured search algorithm (Supporting Information). The main inclusion criterion considered only articles evaluating the effect of anticoagulant poisons in a direct, experimental manner. Publications with titles and abstracts mentioned research of anticoagulants in humans, other animal species, or bats other than *D. rotundus* were excluded. Duplicate articles along with studies that did not fully evaluate the effect of anticoagulants beyond a control method were also excluded. A total of 88 scientific articles were obtained and reviewed. Using the PRISMA method, 12 articles were selected for analysis. The PRISMA method for structured reviews was used to collect the articles, focusing the search on studies evaluating the use of anticoagulants in *D. rotundus* bats (Haddaway et al. 2022). The search equations included specific terms such as ‘common vampire bats’ and used the Boolean operators (AND) and (OR), as well as square brackets [] and quotation marks “” to optimise the precision of the results. Pre‐defined exclusion criteria were then applied to filter and retain only information relevant to the review (Figure 1).

Three study types were included: ‘Laboratory’, ‘Ranch/Field’ and ‘Cave’. The ‘Laboratory’ study type tested *D. rotundus* individuals in chambers. The ‘Ranch/Field’ study type evaluated cattle inside pens. The ‘Cave’ study type observed *D. rotundus* colonies in their natural roosts. Studies were further divided based on whether application of the anticoagulant was ‘Direct’ or ‘Indirect’. Direct application included mainly clinical studies that applied anticoagulant compounds to the skin of *D. rotundus* and administered orally by gavage. Indirect application referred to anticoagulant applied to cattle or the environment. The impact of anticoagulant poison use to control RABV transmission in cattle was evaluated based on its effect on RABV incidence and prevalence.

Using a structured search algorithm, metadata was obtained from Scopus for bibliometric analysis (Supporting Information). The inclusion criteria were articles of different types and objectives researching the use of anticoagulants for the control of *D. rotundus* . Metadata was interpreted using software VOSviewer (version 1.6.19), the R package (4.2.2, 2022‐10‐31 ucrt) Bibliometrix and the web application included in this package Biblioshiny to synthesise the bibliometric indicators (Supporting Information; Aria and Cuccurullo 2017). The software analyses pre‐defined bibliometric indicators (e.g., Citation Index) based on quantitative data from scientific publications between 1972 and 2023 (Aria and Cuccurullo 2017).

Specific bibliometric indicators were identified based on their relevance for understanding research trends and impact. The selected indicators included word co‐occurrence, author co‐occurrence, country co‐occurrence, organisation co‐occurrence, annual average number of publications and annual average number of citations. These indicators were chosen to provide a global view of the research landscape, including thematic trends, collaboration patterns and the impact of published work.

Bibliometric indicators were visualised with co‐occurrence maps and percentage plots. A co‐occurrence map is a visual matrix that shows which terms are frequently used together in a text and how they are related to each other (Galvez 2018). This technique is used to identify thematic structures in scientific texts (Galvez 2018). Percentage graphs were used to represent a scientific evaluation metric with the goal of observing the relevance of research time, such as its productivity, impact factor and citation analysis (Galvez 2018). The bibliometric indicators chosen were co‐occurrence of words, co‐occurrence of authors, co‐occurrence of countries, co‐occurrence of organisations, average annual publication and average annual citation.

## 3 Results

**FIGURE 2:**
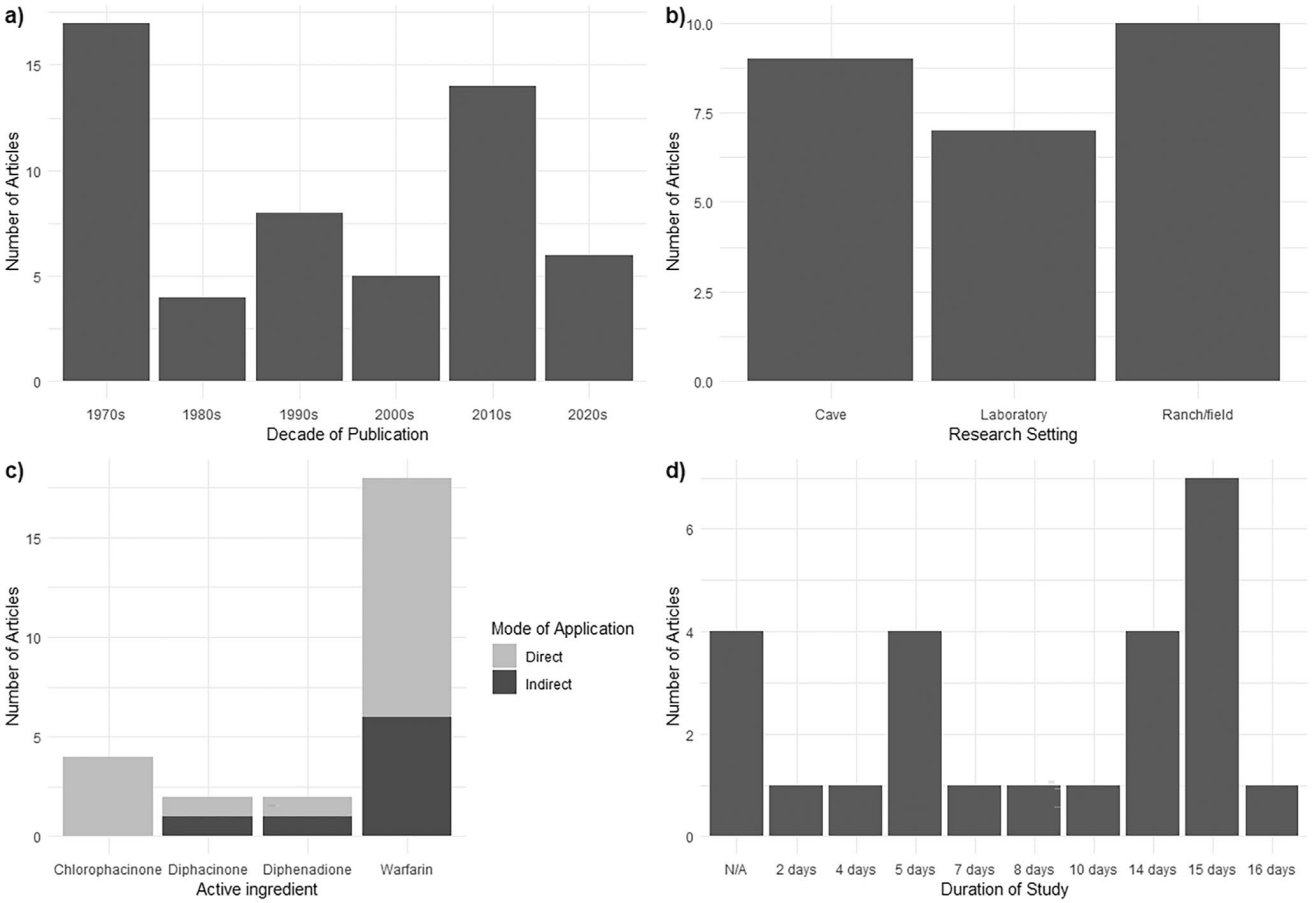
Variable summaries identified in the articles analysed. (a) Number of publications retrieved (*n* = 54) grouped by decade, from 1970 to 2020. (b) Frequency of study type including ranch/field, laboratory and cave. (c) Frequency of active ingredients (warfarin, diphenadione, diphacinone and chlorophacinone) and their modes of application. (d) Time, measured in days, from applying the treatment to observing the results.

Of the 54 articles reviewed, separated by decade, 31.48% were written between 1970 and 1979, 7.41% between 1980 and 1989, 14.81% between 1990 and 1999, 9.26% between 2000 and 2009, 25.93% between 2010 and 2019 and 11.11% between 2020 and 2021 (Figure 2a). Approximately 48% of articles retrieved and reviewed focused on controlling rabies in cattle and bats or controlling *D. rotundus*. Due to the absence of an empirical evaluation of anticoagulants providing quantitative or qualitative data on their use, 26 articles were excluded from the analysis. Quantitative and qualitative information was found in 51.8% (*n* = 28) of the reviewed articles published between 1970 and 1990. A total of 16 articles could not be accessed. Finally, 12 articles were included in the final analysis, describing a total of 26 experimental and field studies on the use of anticoagulants.

Of the 26 experimental studies described in the final 12 articles, 27% were ‘laboratory’ type studies, while 38.6% were conducted in ‘ranch/field’ settings. In addition, 34.6% of the studies were classified as ‘cave’ type (Figure 2b). Warfarin was the most used anticoagulant agent (68%), followed by chlorophacinone (16%), diphacinone (8%) and diphenadione (8%) (Figure 2c). The length of time to observe the desired treatment effect ranged from 2 to 16 days, with 15 days being the most reported period in the studies (Figure 2d).

**FIGURE 3:**
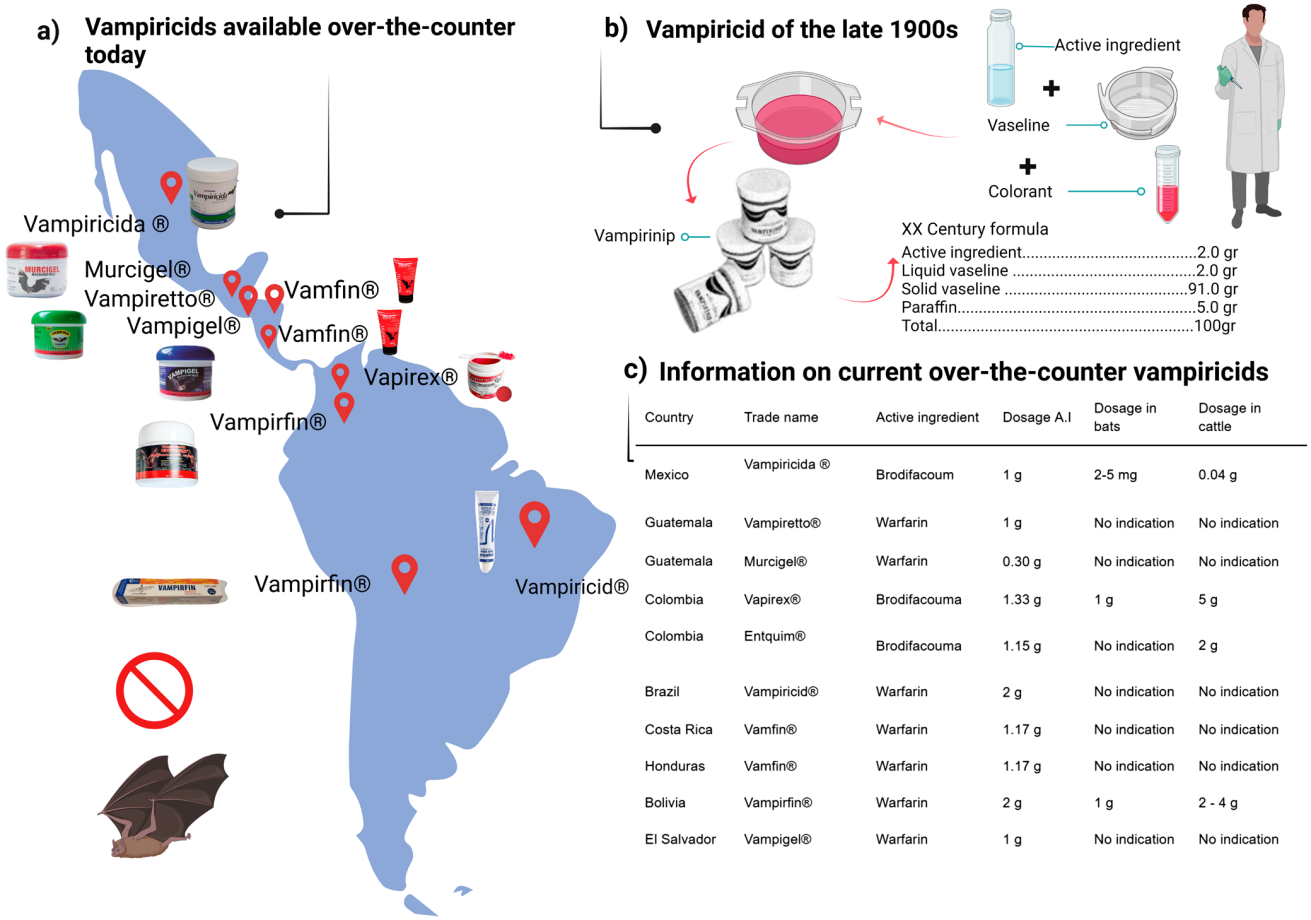
Currently available anticoagulant products. Conceptual diagram explaining the commercial anticoagulant‐based products for killing *D. rotundus* and their active ingredients currently available. (a) Anticoagulant‐based products currently available for the control of *D. rotundus* . Red markers indicate the country of origin of the registered trademarks, the trade name with the symbol (Registered) and a picture of the product. (b) Vampiricid of the late 1900s. The manufacturing process of the first formulated anticoagulant‐based product known as vampiricid (Vampirinip) using an active ingredient, petroleum jelly and red dye is presented. An old photograph of the product is included, as well as a summary table of the formula and composition adapted from Flores‐Crespo and Morales Ruiz (1975). (c) Information on current anticoagulant products known as vampiricids. The table provides details by country of currently available anticoagulant‐based products, including trade name, the active ingredient, the dosage of the active ingredient and dosage for bats and cattle according to the product's indications.

‘Direct’ application was used in 68% of the studies, while ‘indirect’ application was used in 32% of the studies. Direct application included two application methods, dorsal and dorsal‐thoric applications. Anticoagulant dosage varied from 10 mg/mL to 50 mg/1.5 mL (active ingredient/vaseline) representing 2–5 g/bat. A less common direct method was oral gavage in cattle whose reported dose was 0.91 mg/kg (Figure 3).

**FIGURE 4:**
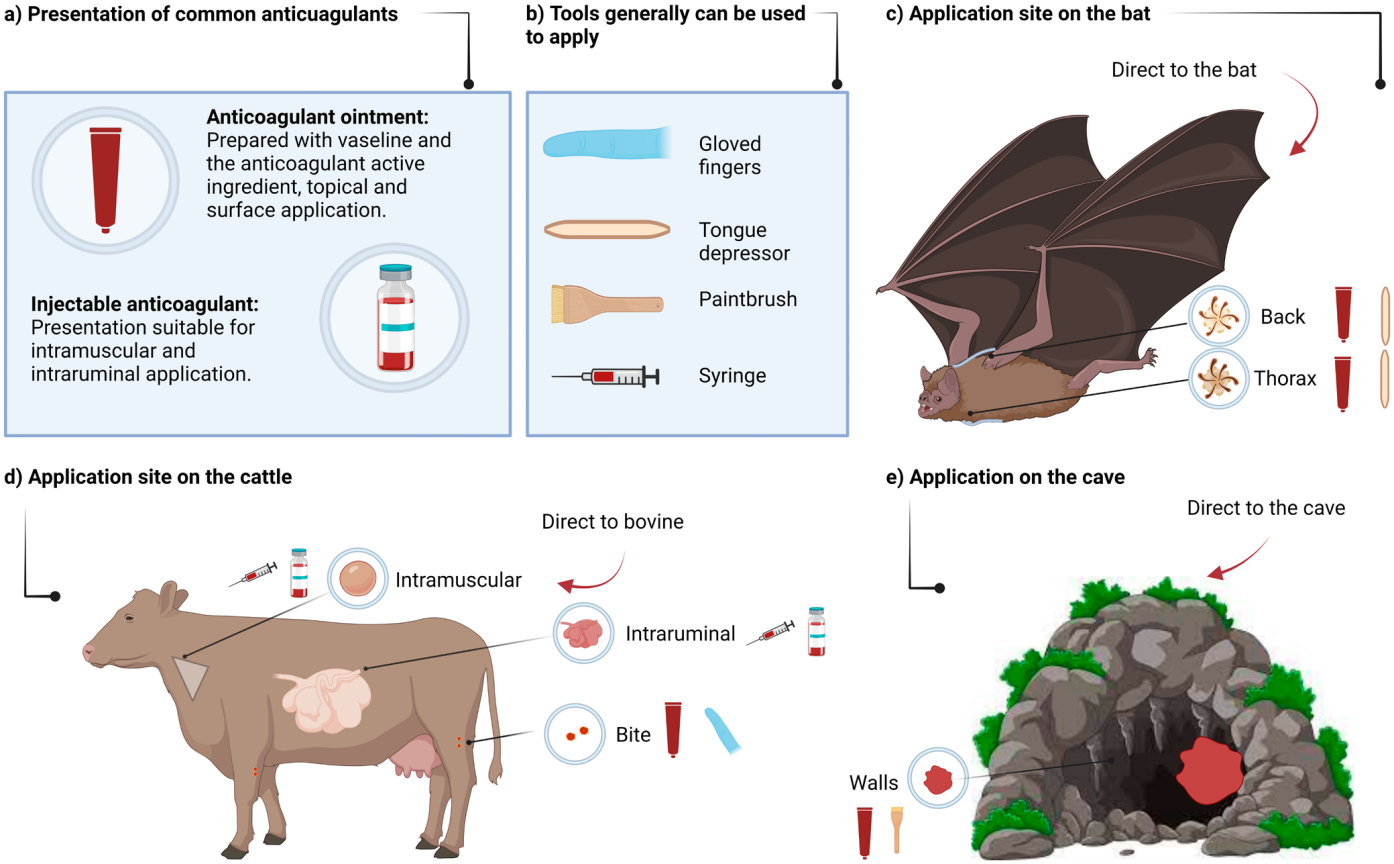
Forms of application of vampiricids products. (a) Two vehicles of the anticoagulants‐based products are used: Ointment and injection. (b) Four tools were generally used for the application of the anticoagulant‐based vampiricid, accompanied by a representative illustration. (c) The application areas on bat individuals where the anticoagulant should be applied are indicated, together with an illustration of the product presentation and its application tool. (d) Cattle‐mediated anticoagulant‐vampiricids are applied to areas of the bovine body, accompanied by an image of the product presentation and its application tool. (e) The indirect application of the anticoagulant‐based product to the walls of the stall is described, accompanied by a picture of the product and its application tool.

Indirect application of anticoagulant‐based products was done by applying the mixture to cave walls, *D. rotundus* bites on cattle and internal intramuscular or intraruminal injections in cattle (Figure 4). For indirect application by injection, the dose of the product varied between 1 and 5 mg/km of live weight of cattle. In the case of wound application, the dosage varied from 10 mg/mL to 50 mg/1.5 mL of active ingredient/vaseline or 2–5 g/wound. Only one cave‐wall application study indicated the dose used (50 mg of the active ingredient in 1.5 g of petroleum jelly) with amounts of product correlated with the treated wall (Table 1).

**TABLE 1:** Treatment information and treated individuals.

Mode of application	Site	Dosage	Treated			Number of individuals		Time	Effects		Effectiveness
			Quantity	Sex	Age	Before	After		PDI*	DBR**	
Treatment information and individuals treated with Warfarin by mode, site, and dosage of application											
Direct	Back	10 mg/g	23	N/A	N/A	245	13	16 days	N/A	N/A	94.7%
		10 mg/mL	1	N/A	N/A	20	0	5 days	N/A	N/A	100%
		N/A	150	N/A	N/A	N/A	N/A	N/A	N/A	N/A	N/A
		10 mg/mL	20	N/A	N/A	20	N/A	15 days	N/A	N/A	96.4%
		10 mg/1 mL	546	313 males 233 females	Scrotale 141 Not scrotal 171 Puppy bat 1 Not pregnant 185 Pregnant 44 With puppy bat 2 Puppy bat 2	546	N/A	7 days	10,920	N/A	N/A
	Back/chest	10 mg/mL	N/A	N/A	N/A	N/A	N/A	10 days	N/A	N/A	N/A
		10 mg/mL	1	N/A	N/A	20	0	5 days	20	N/A	100%
		10 mg/mL	20	Female/Male	N/A	250	6	8 days	81	N/A	32%
		20 mg/2 mL	20	N/A	N/A	37	N/A	14 days	N/A	N/A	96.4%
	Walls	N/A	N/A	N/A	N/A	800	350	15 days	N/A	N/A	55%
		N/A	N/A	N/A	N/A	N/A	N/A	10 days	N/A	N/A	N/A
		N/A	N/A	N/A	N/A	N/A	N/A	15 days	N/A	N/A	N/A
Indirect	Intramuscular	4 mg/kg PV	56	N/A	N/A	56	12	5 days	44	N/A	78.5%
		5 mg kg PV		N/A	N/A	N/A	N/A	15 days		1157	96.30%
	Bite	10 mg/mL	10	N/A	N/A	10	0	4 days	10	N/A	100%
		10 mg/mL	NA	N/A	N/A	N/A	N/A	15 days	N/A	N/A	87.2%
		10 mg/g	NA	N/A	N/A	N/A	N/A	14 days	232	20	66.70%
		10 mg/mL	NA	N/A	N/A	N/A	N/A	N/A	N/A	N/A	N/A
Treatment information and individuals treated with Chlorophacinone by mode, site and dose of application											
Direct	Back	50 mg/1.5 mL	1	N/A	N/A	20	2	14 days	18	N/A	90%
		50 mg/1.5 mL	88	N/A	N/A	91	4	15 days	87	0.02	95.45%
		50 mg/1.5 mL	106	N/A	N/A	2120	1	14 days	2153	N/A	101%
	Walls	50 mg/1.5 g	N/A	43 males and 26 females	158 adults and 25 juveniles	183	12	15 days	149	N/A	81.4%
Treatment information and individuals treated with Diphacinone by mode, site and dose of application											
Indirect	Intraruminal	1 mg/kg PV	N/A	N/A	N/A	N/A	N/A	N/A	N/A	N/A	N/A
Direct	Back	5 mg	N/A	N/A	N/A	N/A	N/A	N/A	N/A	N/A	N/A
Treatment information and individuals treated with Difenadione by mode, site and dose of application											
Direct	Probe	0.91 mg/kg	N/A	Females/male	N/A	N/A	N/A	2 days	N/A	N/A	N/A
Indirect	Intramuscular	4 mg/kg PV	56	N/A	N/A	56	14	5 days	42	N/A	N/A

*Note:* Out of these data, three studies indicated sex and one indicated age. In the indirect application, the dose variable was not frequently recorded. PDI* (number of deceased individuals) and DBR** (decreased bite rate). Ref. (Almeida et al. 2002; Piccini, Freitas, and Souza 1985; Said 1973; Linhart, Flores‐Crespo, and Mitchell 1972; Flores‐Crespo et al. 1979; Flores‐Crespo, Ibarra, and De Anda López 1976; Betancur Hurtado, Calderón Rangel, and Rodríguez 2016; Gonzalez and Mitchell 1976; Flores‐Crespo and Said Fernández 1977; Flores‐Crespo and Morales Ruiz 1975; Flores‐Crespo 1978).

The number of *D. rotundus* treated with an anticoagulant‐based ointment among studies ranged from 1 to 546 individuals. Characteristics such as sex and age were recorded in three studies (Table 1). The most frequent study duration was 15 days, with a range of 2–16 days (Figure 2d).

The number of live individuals before and after application of an anticoagulant‐based compound was the most common response variable. The sample size of *D. rotundus* individuals observed before treatment ranged from 10 to 800, and after treatment, the observed individuals ranged from 1 to 350 (Table 1). The two response variables reported included counts of number of individuals and bites. Studies recorded between 10 and 10,920 *D. rotundus* fatalities attributed to anticoagulant‐based vampiricid treatments. The number of observed bites on cattle ranged from 0.02 to 1157 (Table 1).

**FIGURE 5:**
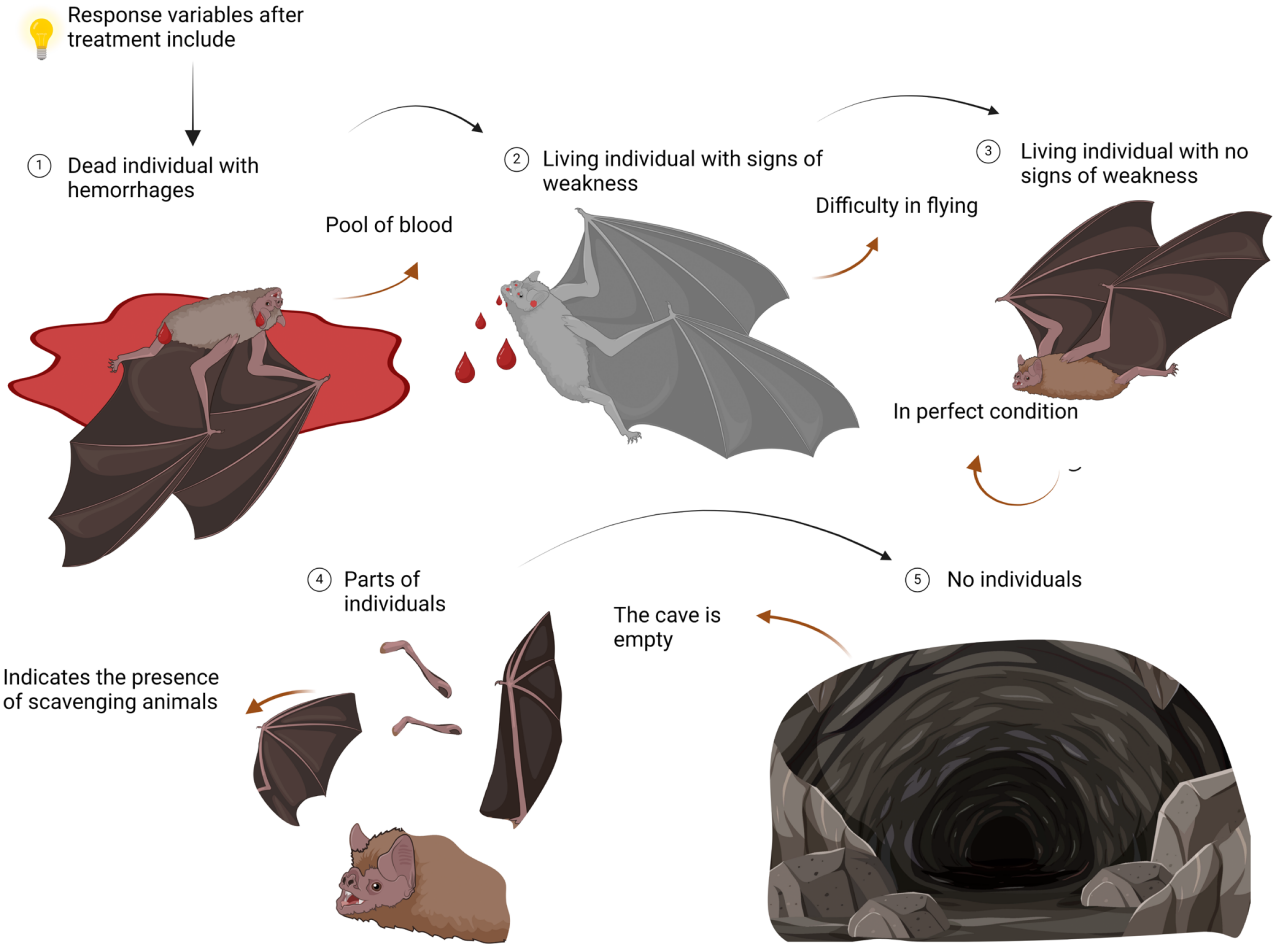
Post‐treatment findings. Scenarios found after anticoagulant treatment in *Desmodus rotundus*. (1) Dead body of bat. (2) Symptomatic bat with aberrant behaviour. (3) Asymptomatic bat. (4) Body parts of bats. (5) Absence of bats.

Studies also reported ‘percent effective’ to summarise the number of live individuals and bites before and after treatment. Direct treatment recorded 32% to 100% effectiveness, while indirect treatments recorded 55% to 100% effectiveness (Table 1). In order to assess treatment effectiveness, post‐treatment results were observed. Figure 5 shows the post‐treatment findings observed in *D. rotundus* after vampiricid application. The resulting observations were identified through evidence of deceased bats (Figure 5.1), bats with visible signs of haemorrhaging due to anticoagulant poisoning (Figure 5.2), bats with no visible signs of poisoning (Figure 5.3), incomplete bat carcasses or bat remains (Figure 5.4) and finally the absence of bats, either live and dead, in the caves.

Regarding the effect on RABV transmission, no study measured or analysed this variable. The results of the bibliometric analysis are six graphs of six bibliometric indicators of interest. Bibliometric results were distributed in two sets.

**FIGURE 6:**
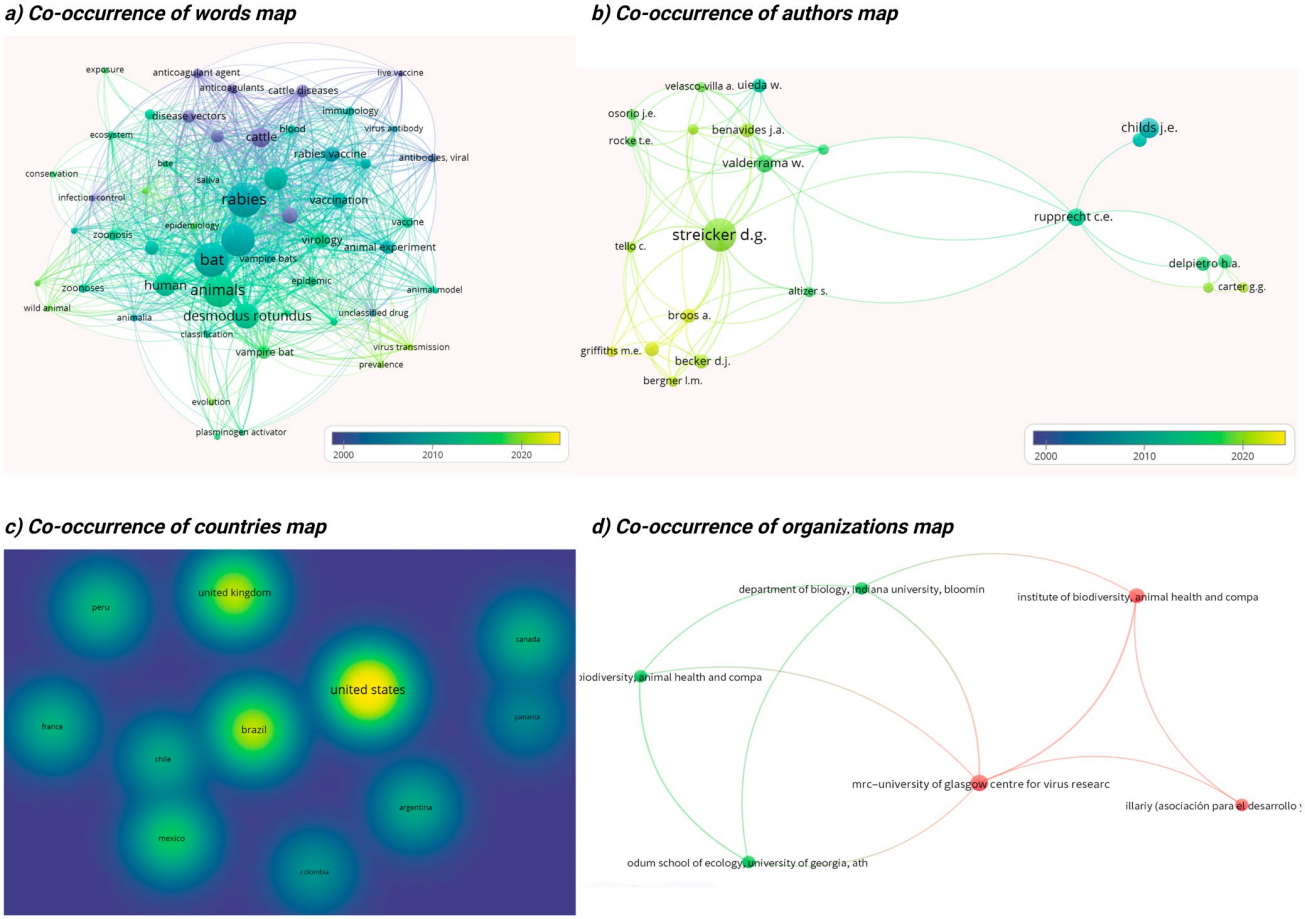
Bibliometric analysis of terms, authors and countries. (a) Word co‐occurrence map. (b) Author co‐occurrence map. (c) Country co‐occurrence map. (d) Organisation co‐occurrence map.

The first set of bibliometric analyses included four co‐occurrence maps. The word co‐occurrence map highlights keywords related to the research topic. The author co‐occurrence map displays the most relevant researchers evaluating anticoagulant use to control *D. rotundus* . The country co‐occurrence map provides information on the geographical distribution of publications, while the organisation co‐occurrence map identifies the main entities involved in these studies. These maps are compiled in Figure 6 containing the word co‐occurrence map (Figure 6a), the author co‐occurrence map (Figure 6b), the country co‐occurrence map (Figure 6c) and the map of organisation co‐occurrences (Figure 6d). The word co‐occurrence map (Figure 6a) identifies that the most relevant and co‐occurring words are ‘rabies’, ‘bat’ and ‘Chiroptera’, while the oldest publications used words related to livestock. Inthe author co‐occurrence map (Figure 6b), the most relevant and recurring authors were identified, with authors such as Streicker and Rupprecht. In the country co‐occurrence map (Figure 6c), the most important and co‐occurring affliated countries are highlighted, with the United States and the United Kingdom being the most represented. Finally, there is no significant density or co‐occurrence among organisations in the map of organisation co‐occurrences (Figure 6d).

**FIGURE 7:**
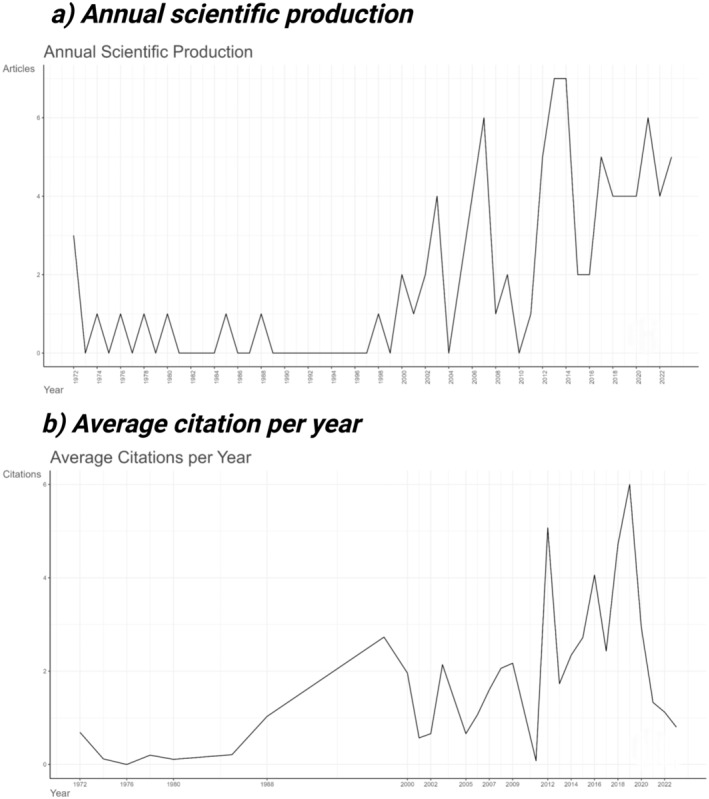
Bibliometric trends of anticoagulants for rabies control. (a) Annual scientific production between 1972 and 2023. (b) Average citation between 1972 and 2023.

The second set of analyses evaluated bibliometric trends related to the use of anticoagulants. This includes a graph of annual scientific publication production between 1972 and 2023, demonstrating the growth of research in this field over time. Then a figure of the average number of citations per year, indicating the impact and relevance of this field of study within the scientific community. Figure 7 contains these analyses within the map of annual scientific production (Figure 7a) and the map of average citations per year (Figure 7b). The map of annual scientific production (Figure 7a) observed that the year with the highest average production was 2013, while the map of average citations per year (Figure 7b) observed that the year with the highest average number of citations per paper was 2019, with 6% (Figure 7; Supporting Information).

## 4 Discussion

Blood loss from *D. rotundus* bites causes economic losses of at least $875 USD per head of cattle (FEDEGAN 2023). Forecasting studies have estimated annual losses of US$7–9.2 million due to RABV transmission in southern Texas alone (Anderson, Shwiff, and Shwiff 2014a). Annual losses of 90,000–100,000 death of cattle heads have been estimated in Mexico alone, where 2769 cattle tested positive for rabies disease between 1997 and 2006 (Acha 1967; Anderson, Shwiff, and Shwiff 2014b). Calculated cattle mortality rates due to *D. rotundus* RABV can be as high as 20% in endemic areas (Prieto and Baer 1972; Martínez‐Burnes et al. 1997; Baer 1991). In Latin America, cattle loss due to *D. rotundus* RABV has been estimated at 100,000–500,000 individuals annually (Swanepoel 2004).

This review explored the use of anticoagulant‐based vampiricids to control *D. rotundus* and *D. rotundus* rabies. The majority of analysed studies were published prior to 2000, with poor to no research on this topic within the last two decades (2000–2020). Among the studies reviewed, warfarin was the most widely used active anticoagulant ingredient and continues to be a widely used product. The number of *D. rotundus* fatalities and bites in cattle was used to determine anticoagulant efficiency. We did not find literature evaluating the effect of anticoagulants to control rabies transmission. These findings imply that current rabies control practices in cattle are guided by references more than 50 to 20 years old.

The deficiency of studies related to the effects of anticoagulant compounds directly tested on *D. rotundus* after 2000 could suggest the implementation of more rigorous animal welfare regulations. Since 1979, animal welfare regulations have been progressively integrated into Latin America's animal research (Russell, Burch, and Hume 1959; Tadich et al. 2020; Heredia Antúnez, Vanda Cantón, and Santillán‐Doherty 2021). Reduced anticoagulant compound research could also be caused by the perceived effectiveness they demonstrate as a RABV control method for agricultural and governmental use (Linhart, Flores‐Crespo, and Mitchell 1972). These studies, however, focused on identifying dosage, application and presentation forms to induce bat mortality. These studies facilitated the development of diverse anticoagulant‐based vampiricid product generations, such as Vampirinip I and Vampirinip II (Flores‐Crespo, Ibarra, and De Anda López 1976), MH‐I and MH‐II (Piccinini, Freitas, and Souza 1985) and Baticide (Moreira et al. 1980; Piccini, Freitas, and Souza 1985).

This study's findings suggest that the literature before 2000 influenced the reduction of *D. rotundus* populations in Latin America. In Colombia, the use of anticoagulant compounds as a control method is actively promoted by the Colombian Agricultural Institute (ICA 2014), in Peru by the National Agricultural Health Service (SENASA 2019) and in Mexico, Guatemala, El Salvador, Honduras, Nicaragua, Costa Rica and Panama by the International Regional Organisation for Agricultural Health (OIRSA 2021; Amezcua Osorio 2021). Application sites and doses reported in the literature mirror methods used by agencies implementing bat and RABV control programs in the region (ICA 2004; Biochem 2019; Wellcopharma 2020).

Of the four active anticoagulant ingredients found in anticoagulant‐based vampiricid products, warfarin, a rodenticide of coumarin origin, decreased in efficacy within rodents during the 1970s. The reduction of warfarin efficiency led to the creation of superwarfarin rodenticides, such as chlorophacinone and diphacinone of indandione origin (Fisher et al. 2019). Currently, the Colombian government uses anticoagulant‐based products, such as Vapirex G or Entquim, which include second‐generation rodenticides such as brodifacouma (ICA 2014; ICA 2022). In Mexico, the product used, Vampiricida, contains bromadiolone as its active ingredient (Biochem 2019). Anticoagulant‐based products in Guatemala (Vampiretto, Murcigel), Brazil (Vampiricid), El Salvador (Vampigel), Costa Rica and Honduras (Vamfin) still use warfarin as their primary active ingredient (Wellcopharma 2020; Mi Agro Shop 2023; MSD Saúde Animal Brasil 2023; Alcames Laboratorios 2024; REINSA 2020; El Finquero 2023a; Laboratorios LLaguno 2023b).

Direct application of anticoagulant to *D. rotundus* bats was the most common mode of application recorded. Direct application may derive from observations of *D. rotundus* feeding and grooming behaviour (Mitchell et al. 1970). Only 40% of *D. rotundus* individuals made sufficient body contact with cattle to receive the topical treatment (Mitchell et al. 1970). A 1974 study found that bat‐cattle contact varied with cattle body movement, temperament and posture (Mitchell et al. 1970; Flores‐Crespo, Fernández, et al. 1974). Less nervous cattle breeds, such as dairy cattle, had a higher percentage of bat contact (85%) compared to beef breeds Charolais (30%) and Brahman (35%) (Mitchell et al. 1970; Flores‐Crespo, Burns, and Said Fernández 1974). As such, direct application to individual bats could offer higher exposure to entire colonies.

*Desmodus rotundus* grooms by age groups, with adult individuals primarily going outdoors to feed and then groom. Differences during grooming hierarchy may generate biased targeting of specific bat groups. Adult bats the main vehicles of the product and, subsequently, the main mortalities of anticoagulant poisoning (Streicker, Lemey, et al. 2012; León et al. 2021). Age is linked to immune status and seroprevalence to RABV. Accordingly, juvenile *D. rotundus* shows higher RABV seroprevalence and lower exposure to anticoagulant poisoning (Streicker, Recuenco, et al. 2012). When young *D. rotundus* males reach sexual maturity, they disperse an average of 20 km away to form a new colony, which is linked to higher RABV transmission (León et al. 2021; Streicker et al. 2016).

Individual‐specific traits, such as age and sex, were generally not considered in anticoagulant product studies. Additionally, although rabid animals tend to groom less, the behaviour of rabid individuals against healthy ones was not included in studies (Blackwood et al. 2013). The effect duration and frequency of anticoagulant product application on *D. rotundus* colonies have rarely been evaluated (Mitchell et al. 1970). Observations of repopulation time within *D. rotundus* colonies determined that they could also be influenced by geographic location and environmental variation (Mitchell et al. 1970, 1971).

Our review revealed that after the use of anticoagulant vampiricids, bites in cattle reduced and *D. rotundus* mortality increased. Therefore, the use of anticoagulants‐based vampiricids could be an effective method to prevent *D. rotundus* attacks. Nevertheless, some studies have found conflicting results regarding the survival of *D. rotundus* colonies after treatment, reporting both increases and decreases in survival rates over the years (Almeida et al. 2002; Streicker, Lemey, et al. 2012). Rabies disease outbreaks have been recorded in areas where *D. rotundus* anticoagulant‐based vampiricid control has taken place for multiple days (Rocha and Dias 2020; Streicker et al. 2019; Viana et al. 2023). Therefore, it is likely that the transmission of RABV is not related to the population density of *D. rotundus* alone (Johnson, Aréchiga‐Ceballos, and Aguilar‐Setien 2014; Benavides, Valderrama, and Streicker 2016).

*Desmodus rotundus* uses a variety of natural and artificial shelters, including hollow trees, tunnels, mines and caves (Marín et al. 2008; Mialhe 2013; Greenhall, Joermann, and Schmidt 1983). Some of these refuges are permanent and large, housing colonies of females and young, while others are temporary and used for resting or socialising (Mantovan et al. 2022; Marín et al. 2008). This change in refuge use is one of the reasons why *D. rotundus* moves between different areas (Mantovan et al. 2022; Marín et al. 2008).

Refuge movement in *D. rotundus* is related to male behaviour, where a few alpha males dominate multi‐female colonies, while non‐dominant or ‘single’ males seek to mate and form their own colonies (Marín et al. 2008; Mialhe 2013). Shelter shifting in *D. rotundus* is also related to their foraging strategy, where they seek to locate nearby food sources such as livestock (Streicker, Recuenco, et al. 2012; Moya, Pacheco, and Aguirre 2015; Lee, Papeş, and Van den Bussche 2012). Studies suggest that colony size and concentration are directly dependent on the density of available cattle (Streicker, Lemey, et al. 2012; Moya, Pacheco, and Aguirre 2015; Lee, Papeş, and Van den Bussche 2012). When cattle move or change area, *D. rotundus* follows these movements and seeks new refuges close to its food sources (Moya, Pacheco, and Aguirre 2015).

Unnatural *D. rotundus* displacement occurs when colonies are disturbed. Cave treatments attribute the absence of bats to effective mortality caused by application of anticoagulant‐based vampiricid. However, the absence of bats may be due to disturbance of their roosts during the application of the treatment (Blackwood et al. 2013). Factors such as human presence, noise, netting and manipulation of individuals force them to seek new roosts. On the other hand, studies suggest that it is inadvisable to control *D. rotundus* when rabies disease outbreaks are already present, but rather at the onset of attacks (Uieda and Gonçalves de Andrade 2020). Once the virus is already present in the region, culling bats would be ineffective (Uieda and Gonçalves de Andrade 2020; Kraker‐Castañeda et al. 2024). This would leave the roosts empty and attract other bats whose immune status is unknown (Rocha and Dias 2020; Streicker, Recuenco, et al. 2012). Repopulation of empty roosts facilitates the spread, maintenance and transmission of RABV in endemic areas (Corrêa Scheffer et al. 2014; Benavides et al. 2020; Gonçalves, Galetti, and Streicker 2021; Rocke, Streicker, and Leon 2023).

Another response variable was finding the remains of several dead *D. rotundus* individuals (e.g., wings, head and arms) in the study area, suggesting that these bats affected by the anticoagulant paste had been preyed on. This raises the possibility of secondary or indirect poisoning, similar to documented cases of rodenticide use on rodents, where predatory species such as owls and cats, as well as scavengers, may be affected (Walker et al. 2008; Riley et al. 2007; Elliott et al. 2014). In addition, *D. rotundus* share caves with other bat species (Benavides et al. 2020), which increases the risk that anticoagulant‐based vampiricids used could inadvertently poison these other species (Delpietro et al. 2021; Mayen 2003). Efforts to control *D. rotundus* have had a negative impact on other bat species endemic to the Brazilian Cerrado. In fact, a predictive study found that control efforts that use anticoagulant compounds may affect the survival of the bat *Lonchophylla dekeyseri* , which shares roosts with *D. rotundus* (Aguiar, de Camargo, and Portella 2006; Aguiar, Brito, and Machado 2010). This problem is related to the destruction of more than 8000 caves in the region where both bats co‐occur since the 1960s, using explosives and other methods to block bat access (Walker et al. 2001; Aguiar, Brito, and Machado 2010). In addition, landowners in the region have used warfarin‐based vampiricids without regard to the species involved. This approach has put *L. dekeyseri* at risk and dispersed the impact of control measures on *D. rotundus* . Reports of collateral damage from anticoagulant‐based vampiricids use include mortality of fish and diverse bats species (Flores‐Crespo, Fernández, et al. 1974).

Available literature on the use of anticoagulants based *D. rotundus* control use terms linked with methods to reducing the impact of vampire bat RABV on livestock and the economy (Piccini, Freitas, and Souza 1985; Said 1973; Linhart, Flores‐Crespo, and Mitchell 1972; Flores‐Crespo et al. 1979). The most relevant authors, countries and organisations in the vampire‐control literature do not represent countries with vampire bat RABV, with the emblematic case of the United States. The recent interest of such country on the control or rabies may be due to potential risk of RABV spread from Latin America to Texas (Anderson, Shwiff, et al. 2014). Annual production and citation indicators showed a growing research interest in vampire bat rabies, which follows the ‘exponential growth law’ of global literature on a topic doubling every 10–15 years (Tarrío‐Saavedra, Orois, and Naya 2018). Despite this, there were no publications assessing the effectiveness of anticoagulant‐based vampiricids for RABV control (Rocke, Streicker, and Leon 2023).

## 5 Conclusions

Anticoagulants reduce the number of *D. rotundus* bats and bites in cattle, which could likely reduce economic losses by farmers due to cattle loss. Nevertheless, we did not find economic studies to support this hypothesis. No conclusive evidence was found on the use of anticoagulants as a method to successfully control rabies. After more than 50 years of using anticoagulant products to control *D. rotundus* , little is known regarding the actual effect of bat culling on the epidemiology of rabies. Instead, recent literature suggests that culling *D. rotundus* may be counterproductive, increasing RABV transmission, circulation and spread to new areas and the reduction of non‐target bat species.

## Conflicts of Interest

The authors declare no conflicts of interest.

## Supporting information


Appendix S1

## Data Availability

The data that support the findings of this study are available from the corresponding author upon reasonable request.
