# Supplementary material for: Anticoagulants for the Control of the Common Vampire Bat (Desmodus rotundus)
Source: Zoonoses Public Health. 2024 Dec 22;72(2):101–16. doi: 10.1111/zph.13196 (PMC11772909; doi:10.1111/zph.13196)
Supplement: Supplementary file 1 — Appendix S1 [file ZPH-72-101-s001.docx]

**Supplementary material**

**Materials and Methods**

*Selection Criteria*

Inclusion criteria were publications on the use of anticoagulants as a method of controlling Desmodus rotundus, and publications on the component of bat saliva for medical purposes, such as draculin, were discarded. Articles from 1969 to 2022 and from all countries were included. Incomplete articles with missing methodology, results, or only the published abstract and limited access to the full text were excluded, as well as articles that, when fully reviewed, only mentioned the use of these anticoagulants but not the purpose of evaluating their effectiveness, articles that summarized or collected all methods of anticoagulant use, but with informative purposes, were also excluded. Only articles evaluating an anticoagulant product for the control of D. rotundus were selected.

*Information Sources*

The databases selected were Scopus, Web of Science, Science Direct, Scielo, PubMed, CABI, EBSCO, and Google Scholar; the search for information began in August 2022 and ended in August 2023.

*Search Strategy*

The following terms were selected for the search: vampire bats, common vampire bats, Desmodus rotundus, anticoagulant, culling, poisoning, sacrifice, warfarin, chlorophacinone, and brodifacoum. The following query was generated with these terms:

- (“culling vampire bats”)
- ("Desmodus" OR "Desmodus rotundus" OR "common vampire bat") AND ("anticoagulant")
- (“Anticoagulant effectiveness” OR “Anticoagulants effectiveness”) AND (“Desmodus rotundus OR “Desmodus” OR “vampire bats” OR “common vampire bat”)
- (“effective anticoagulants” OR “effective anticoagulant”) AND (“Desmodus rotundus OR “Desmodus” OR “vampire bats” OR “common vampire bat”)

Each equation went through the selected databases to later count the total results and select the articles by title and abstract. The articles selected by abstract and title according to the search were read in full to discard those that were incomplete, could not be found on the Web, were inaccessible, or did not meet the inclusion criteria. Qualitative and quantitative data were processed for each article, including country, city, year, time of study, type of study, active ingredient, method of application, site of application, dose used, number of individuals before and after application, number of specimens treated, waiting time for results, method of evaluation of results, results in humans and in cattle, calculated efficacy, and postmortem findings. These variables were analyzed and visualized in R and R Studio (4.2.2, 2022-10-31 ucrt) using the ggplot2 package.

**Bibliometric analysis**

*Search equation*

Key words such as "vampire bat," "Desmodus rotundus," "anticoagulant," "control," or "rabies" and other synonymous or similar terms were used to formulate the search equation. Boolean operators such as AND and OR were used, as well as search modifiers such as quotation marks and parentheses, to generate a more precise search equation. Equations such as:

- ( "common vampire bat" OR “murciélago vampiro) AND ("control")
- ("Desmodus" OR "*Desmodus rotundus*" OR "common vampire bat") AND ("anticoagulant")
- (“vampire bat”) AND (“anticoagulant”) AND (“control”) AND (“rabies”)

The following search equation was chosen for the bibliometric analysis: *(“vampire bat” OR “common vampire bat” OR “Desmodus rotundus”) AND (“anticoagulant” OR “anticoagulante”) AND (“control” OR “sacrificio” OR “culling”) AND (“rabies” OR “rabia”).*

To obtain a larger number of documents, articles, books, and other types of publications were included. Results from all years and all countries were included. The results were refined by excluding documents on pharmacology and fields not related to the subject of the study. The Scopus and Web of Science databases were searched. Those whose results mentioned other species of bats, documents with pharmacological purposes or the use of anticoagulants for medical purposes in humans were discarded. The equation whose results most closely matched the subject of the study was selected. The database with the highest results was Scopus, compared to Web of Science, whose results were less than 50 documents. We decided to perform the bibliometric analysis on the metadata retrieved from Scopus.

*View Metadata*

Once the metadata were retrieved from Scopus, they were visualized using the VOSviewer software (version 1.6.19), the RStudio package (4.2.2, 2022-10-31 ucrt) Bibliometrix and the web application included in this package Biblioshiny (Aria & Cuccurullo, 2017). VOSviewer was used to generate different co-occurrence maps of keywords, authors, countries, and organizations by loading the retrieved metabase into the software and filtering out data that were not very relevant to the study. These data were visualized by means of graphs and tables on the annual production and average citation of documents on the use of anticoagulants for the control of D. rotundus. The bibliometric indicators chosen for the analysis were year of publication, countries, associations, authors, keywords and average number of citations.
